# The effect of follicular fluid selenium concentration on oocyte maturation in women with polycystic ovary syndrome undergoing in vitro fertilization/Intracytoplasmic sperm injection: A cross-sectional study

**DOI:** 10.18502/ijrm.v19i8.9616

**Published:** 2021-09-09

**Authors:** Seyedeh Mahsa Poormoosavi, Mohammad Amin Behmanesh, Hossein Najafzadeh Varzi, Shabnam Mansouri, Sima Janati

**Affiliations:** ^1^Department of Histology, School of Medicine, Research and Clinical Center for Infertility, Dezful University of Medical Sciences, Dezful, Iran.; ^2^Department of Histology, School of Medicine, Dezful University of Medical Sciences, Dezful, Iran.; ^3^Department of Pharmacology, Faculty of Medicine, Babol University of Medical Sciences, Babol, Iran.; ^4^School of Medicine, Dezful University of Medical Sciences, Dezful, Iran.; ^5^Department of Obstetrics and Gynecology, School of Medicine, Research and Clinical Center for Infertility, Dezful University of Medical Sciences, Dezful, Iran.

**Keywords:** Polycystic ovary syndrome, Oxidative stress, Selenium, In vitro fertilization, Oocyte quality, Follicular fluid.

## Abstract

**Background:**

A high level of free radicals and oxidative substances in women with polycystic ovary syndrome (PCOS) can affect the ovaries through oxidative stress. Antioxidants such as selenium, a vital trace element in human health, can improve the prognosis of PCOS by reducing oxidative stress.

**Objective:**

This study was performed due to the lack of comprehensive information about selenium concentration in follicular fluid and its effect on the oocyte count and quality in infertile women with PCOS.

**Materials and Methods:**

In this cross-sectional study, 78 women with PCOS referred to Umm-al-Banin Infertility Clinic Center, Ganjavian Hospital, Dezful, Iran for in-vitro fertilization from March to November 2019 were enrolled. After ovarian stimulation with the antagonist protocol, the oocytes were retrieved under transvaginal ultrasound in in-vitro fertilization/intracytoplasmic sperm injection cycles, and selenium concentrations were measured in the follicular fluid using an atomic absorption method by spectrophotometer device. Oocyte count and morphology were evaluated using inverted optical microscopy.

**Results:**

There were no significant differences between follicular fluid selenium concentrations in terms of the total number of oocytes and immature oocytes in the metaphase I and germinal vesicle stages. However, a significantly reduced number of metaphase II oocytes was observed at selenium levels < 40 μg/dL (p = 0.001).

**Conclusion:**

Based on our results, low levels of follicular selenium concentration in infertile women with PCOS can reduce the quality and potency of oocyte maturation.

## 1. Introduction

Polycystic ovary syndrome (PCOS), which has metabolic and reproductive outcomes (1), is one of the most common endocrine disorders affecting approximately 7-10% of women of reproductive age worldwide (2, 3). Hyperandrogenemia, high body mass index (BMI), insulin resistance, and other conditions related to PCOS can lead to augmented inflammation and oxidative stress (4).

Human follicle development involves several intraovarian and endocrine activities that initiate an alternation in the intrafollicular microenvironment for optimum conditions for oocyte competency (5).

Assessing the quality and morphology of oocytes is the basis of assisted reproductive techniques (6). Although women with PCOS undergoing in vitro fertilization (IVF) have more oocytes and less need for stimulation with gonadotropins, their oocytes are less capable of growth and are unable to complete meiosis and fertilization, and form a normal embryo (7). However, the genetic and environmental aspects of the complexity of PCOS and how PCOS affects the oocyte development are not yet fully understood (8). Follicular fluid (FF) is the net product of the shift of plasma ingredients to follicles and the secretory action of theca cells (5), as well as the blood-follicle fence of the granulosa cells, which plays vital role in oocyte development and maturation. In fact, the development and viability of oocytes are affected by certain compositions of FF (6) that can reflect any alterations in ovarian cell secretory processes and changes in the plasma components due to pathological conditions. Thus, FF improves the oocyte developmental capacity and ovulation and also acts as a medium for the connection between follicular cells and oocytes through follicular growth (9). Studies have shown higher levels of reactive oxygen species (ROS) in the FF of women with PCOS and endometriosis (5, 10). Coskun and colleagues found a positive association between a decrease in oxidative stress and an increase in oocyte maturation in infertile women with PCOS. They concluded that antioxidants could improve the prognosis of PCOS by decreasing oxidative stress (11).

Selenium is an essential element in the human body that, as an effective antioxidant in selenoproteins, supports redox activities (12, 13). Therefore, selenium may affect reproductive outcomes, inflammatory biomarkers, and oxidative stress in women with PCOS (12), which could be attributed to its inhibitory effects on pro-inflammatory cytokines, reactive oxygen, and nitrogen species (14). A study showed that decreased selenium levels of serum and FF in women undergoing IVF compete with nonpregnant women (15). However, the relationship between selenium levels and fertility parameters has not yet been established (16).

Coskun and colleagues showed that decreased plasma levels of selenium and its negative association with hormonal levels (LH, total testosterone) in patients with PCOS may indicate that selenium has a role in the pathogenesis of metabolic disorders and oxidative stress associated with PCOS (11).

Considering that examining the elements present in the FF is the most accessible way to measure oocyte status, this study aimed to evaluate the selenium concentration of FF in women with PCOS undergoing IVF/Intracytoplasmic sperm injection (ICSI) cycles and its effect on the oocyte count and quality in these women.

## 2. Materials and Methods

### Study design and participants 

This cross-sectional study was conducted with 78 infertile women with PCOS aged 20-45 yr who were referred to the Umm-al-Banin Infertility Clinic Center, Ganjavian Hospital, Dezful, Iran for IVF/ICSI from March to November 2019. Participants were randomly selected via the block randomization method. The PCOS diagnosis was based on fulfilling at least two out of the following three Rotterdam criteria: (i) clinical signs or biochemically excessive androgen level, (ii) oligomenorrhea and/or anovulation, and (iii) morphology of ovaries in sonography termed as 12 or more small follicles (17).

The inclusion criteria of the study were included women aged 20-40 yr diagnosed with PCOS and with serum levels of follicle-stimulating hormone (FSH) < 10 IU/L on the third day of the cycle.

The exclusion criteria were other causes of infertility, endometriosis, metabolic disorders, diabetes mellitus, cigarette smoking, consumption of antioxidant drugs or supplements containing selenium three months prior, empty follicle and ovarian hyper stimulation syndrome.

Data including the age of women and their spouses', duration of infertility, menstrual cycle's striation, and the history of taking any supplement such as selenium in the past three months were collected by questioning the individuals. In addition, height, body weight, and BMI of the women were measured.

### Ovarian stimulation, oocyte retrieval, and FF extraction

Controlled ovarian stimulation was performed using the antagonist protocol. All participants used low-dose oral contraceptive pills (OCP LD; contains 0.3 mg Norgestrel + 30 μg Ethinyl Estradiol, Aburaihan Pharmaceutical Co., Tehran, Iran) which were started on the second day of the pre-gonadotropin cycle and continued until menstruation. After the menstruation onset, the stimulation protocol was started with recombinant-FSH (Gonal-F, Serono Co., Italy) from the second day of the menstrual cycle.

The initial dose of gonadotropin was 150-300 mIU/d, based on the women's age, body weight, and previous ovarian response. The monitoring was performed on the seventh or eighth day of stimulation and the dose of gonadotropin was altered according to the serum estradiol measurement and ovarian response, which were assessed by consecutive vaginal ultrasound.

Once the leading follicles attained a diameter of 14 mm, 0.25 mg of cetrorelix (Merck-Serono, Germany) was added subcutaneously and repeated daily until the day of human chorionic gonadotropin (HCG) administration. 6500 IU of recombinant HCG vial (rhCG) (Ovitrelle, Merck-Serono, Germany) was administered subcutaneously until at least three follicles reached ≥ 18 mm in diameter. The ovum retrieval was performed vaginally, using ultrasound and precise aspiration 36 hr after rhCG.

After retrieval, oocytes were collected from the FF and washed with a G-MOPS medium (Vitrolife, Sweden) and then covered with mineral oil (Ovoil; Vitrolife, Sweden) and incubated in a culture medium (GIVF-plus; Vitrolife, Sweden) for 2 hr at 37°C, 6% CO${}_{2}$ and 5% O${}_{2}$. Cumulus cells around the oocyte were separated manually after 30 sec exposure in a Hyase medium having 80 IU/mL of hyaluronidase (Vitrolife, Sweden). The nuclear state of denuded oocytes was then determined. The oocytes were graded into the following three classes via an inverted microscope (Olympus, Japan): "Metaphase II (MII) - presence of the first polar body; Metaphase I (MI) - absence of the first polar body; and germinal vesicle breakdown (GV) - presence of a clearly defined germinal vesicle containing the typical prominent nucleolus and degenerated oocytes (18)". The oocytes were then inseminated. Injected oocytes were incubated in the same culture medium. The oocytes were checked 16-18 hr following injection to determine the presence of pronuclei using a Nikon inverted microscope (Olympus, Japan).

To minimize any probable interfering, blood-free FF samples were used for the measurements using photometric assay. To precipitate blood cells and remove cellular components, 1 ml of the harvested FF was centrifuged at 3,000 rpm for 10 min. At the final stage, only the supernatant of the blood-free samples was loaded into cryovials and stored at -70°C until assayed (19).

### Selenium measurement 

Samples were diluted with nickel nitrate and nitric acid based on Campillo's method. The selenium levels of the FF were measured by Atomic Absorption (Varian 240 FS, USA). Measurements were performed using a 1-nm wide tube and atomic absorption of 196 nm. Argon was used as an inert gas at a flow of 150-250 ml/min (20).

### Ethical considerations

The study was approved by the Ethics Committee of Dezful University of Medical Sciences, Dezful, Iran (Code: IR.DUMS.REC.1397.006). Written consent was obtained from all participants before the study.

### Statistical analysis 

All of the statistical analyses were performed using the Statistical Package for the Social Sciences software (SPSS version 22.0 for Windows; SPSS Inc., Chicago, USA). The results were expressed as mean ± SD. The differences between the mean oocyte quantities were assessed by one-way ANOVA and LSD tests. P < 0.05 were considered statistically significant.

## 3. Results 

In total, 741 oocytes from 78 women with PCOS were collected. The mean ± SD of the total retrieved oocytes and of the MII, MI, and GV oocytes were estimated as 9.61 ± 3.26, 7.68 ± 4.12, 2.87 ± 1.04, and 1.33 ± 0.87, respectively.

The total number of oocytes was significantly higher in women aged < 40 yr than in older women (p = 0.011), and MII oocytes was significantly higher in women aged < 35 yr than in older women (p = 0.008). In women aged ≥ 40 yr, the total number of oocytes and MI oocytes was lower than in women < 40 yr and the number of GV oocytes was significantly higher in 35-yr-old women than in younger ones (Table I).

Women with PCOS who had a BMI ≥ 30 kg/m${}^{2}$ had significantly fewer total oocytes, MI, and MII oocytes compared with women who had a lower BMI. In contrast, there was no significant difference in the number of GV oocytes. Participants with female-factor infertility or both male and female factors, had significantly fewer total oocytes and MI and MII oocytes compared to participants with male-factor infertility. No association was found between immature GV oocytes and infertility causes (Table I).

A duration of < 5 of infertility years was associated with a significantly higher in number of immature GV oocytes compared with longer infertility duration. However, in women with an infertility history of 5-10 yr, a significantly smaller total number of oocytes was observed than in other women. Women with an infertility duration of > 10 yr showed a significantly lower number of MII mature oocytes than women with shorter infertility duration. However, no significant relationship was found between the number of immature MI oocytes and the infertility duration (Table I).

The mean count of MII oocytes was significantly lower in the women with an FF selenium concentration < 40 µg/ml than in the women with higher concentrations (p = 0.001). However, no significant association was observed between the selenium concentration in FF and the count of the total oocyte, the MI oocytes or the GV oocytes (p = 0.98, 0.13, and 0.22, respectively).

Although the highest total oocyte count was seen at selenium concentrations > 60 µg/ml, the difference in the total oocyte count was not significant. The number of MII oocytes in concentrations < 40 µg/ml was significantly lower than in higher concentrations (Table II).

**Table 1:** Comparison of the mean number of oocytes at different oocyte maturation phases


**Variables**		**Total number of oocytes**	**MII**	**MI**	**GV**
**Women's age (yr)**					
	**25-30**	10.24 ± 3.5${}^{a}$	4.2 ± 1.33${}^{a}$	4.22 ± 1.2${}^{a}$	1.9 ± 0.4${}^{b}$
	**31-34**	11.5 ± 3.5${}^{a}$	4.17 ± 1.8${}^{a}$	5.5 ± 0.7${}^{a}$	2.4 ± 0.9${}^{b}$
	**35-39**	10.33 ± 3.4${}^{a}$	2.24 ± 0.9${}^{b}$	4.11 ± 1.1${}^{a}$	3.6 ± 1.4${}^{a}$
	**40-44**	7.1 ± 1.3${}^{b}$	2.54 ± 0.4${}^{b}$	2.7 ± 0.5${}^{b}$	3.28 ± 0.6${}^{a}$
	**≤45**	6.97 ± 1.9${}^{b}$	2.27 ± 0.8${}^{b}$	2.21 ± 0.4${}^{b}$	3.24 ± 0.6${}^{a}$
	**p-value **	0.011	0.008	0.02	0.004
**Women BMI (kg/m${}^{2}$)**					
	**< 25**	10.31 ± 2.7${}^{a}$	3.14 ± 1.1${}^{a}$	4.27 ± 1.2${}^{a}$	2.33 ± 0.4${}^{a}$
	**25-29.99**	11.21 ± 3.23${}^{a}$	3.7 ± 1.2${}^{a}$	4.5 ± 1.13${}^{a}$	2.74 ± 0.71${}^{a}$
	**30-34.99**	5.66 ± 2.9${}^{b}$	1.94 ± 0.7${}^{b}$	2.66 ± 0.9${}^{b}$	2.41 ± 0.83${}^{a}$
	**≤ 35**	5.8 ± 2.4${}^{b}$	2.21 ± 0.94${}^{b}$	1.12 ± 0.5${}^{b}$	2.5 ± 0.75${}^{a}$
	**p-value**	0.002	0.011	0.003	0.17
**Cause of infertility (yr)**					
	**Male factor**	9.55 ± 3.5${}^{a}$	4.74 ± 1.4${}^{a}$	3.25 ± 1.3${}^{a}$	1.27 ± 0.4${}^{a}$
	**Female factor**	4.47 ± 3.9${}^{b}$	2.56 ± 0.6${}^{b}$	1.42 ± 0.4${}^{b}$	1.27 ± 0.4${}^{a}$
	**Both**	4.21 ± 3.7${}^{b}$	2.32 ± 1.03${}^{b}$	1.77 ± 0.7${}^{b}$	1.81 ± 0.6${}^{a}$
	**p-value**	0.011	0.002	0.03	0.26
**Infertility duration (yr) **					
	**< 5**	10.25 ± 3.7${}^{a}$	4.17 ± 1.6${}^{a}$	2.55 ± 0.8${}^{a}$	3.42 ± 1.1${}^{a}$
	**5-10**	6.64 ± 4.3${}^{b}$	3.21 ± 1.6${}^{a}$	2.14 ± 0.9${}^{a}$	1.14 ± 0.7${}^{b}$
	**> 10**	5.5 ± 2.98${}^{b}$	1.7 ± 0.8${}^{b}$	2.98 ± 0.94${}^{a}$	1.22 ± 0.44${}^{b}$
	**p-value**	0.016	0.03	0.20	0.012
All data presented as Mean ± SD (one-way ANOVA and LSD tests was used for all parameters, p < 0.05), ${}^{a,b}$Significant differences at p ≤ 0.05. BMI: Body mass index, MI: Metaphase I, MII: Metaphase II, GV: Germinal vesicle					

**Table 2:** Comparison of the mean distribution of oocytes between different levels of follicular fluid selenium concentration


**Selenium concentration (µg/ml)**	**Total number of oocytes**	**MII**	**MI**	**GV**
**20-30**	9.25 ± 1.1${}^{a}$	2.54 ± 0.2${}^{b}$	5.47 ± 0.7${}^{a}$	1.23 ± 0.3${}^{a}$
**31-40**	9.74 ± 3.13${}^{a}$	2.33 ± 0.5${}^{b}$	5.37 ± 1.34${}^{a}$	1.66 ± 0.7${}^{a}$
**41-50**	10.5 ± 2.6${}^{a}$	5.37 ± 1.66${}^{a}$	4.47 ± 0.2${}^{a}$	1.37 ± 0.5${}^{a}$
**51-60**	9.13 ± 3.14${}^{a}$	5.38 ± 0.73${}^{a}$	4.45 ± 1.54${}^{a}$	1.41 ± 0.74${}^{a}$
**61-70**	10.64 ± 3.26${}^{a}$	5.32 ± 1.43${}^{a}$	4.64 ± 1.5${}^{a}$	0.87 ± 0.33${}^{a}$
**p-value**	0.98	0.001	0.13	0.22
All data presented as Mean ± SD (one-way ANOVA and LSD tests was used for all parameters, p < 0.05), ${}^{a,b}$Significant differences at p ≤ 0.05. MI: Metaphase I, MII: Metaphase II, GV: Germinal vesicle				

## 4. Discussion

This study was designed to evaluate the effect of FF selenium concentration on oocyte quantity and maturation in infertile women with PCOS. According to our results, there was a negative effect on oocyte maturation and the number of total oocytes and MII oocytes at low concentrations of selenium.

Intra-follicular paracrine signaling plays an important role in follicular growth and development (21). The components of FF can be changed directly by hormonal, paracrine, and autocrine signalling pathways, as well as indirectly by systemic disorders (22). Changes in follicular levels of antioxidants, hormones, and metabolites have been observed at various stages of follicular development (23). Studies have shown that ROS is required for ovulation in mice, but that excessive levels of ROS can cause deflections in microtubules and the chromosomal arrangement of MII meiotic spindles (24, 25). It seems that changes in the metabolites components of FF can affect oocyte quality, early embryo development, and subsequent pregnancy (22). On the other hand, the cumulus cells play an important role in oocytes maturation by synthesizing and transferring glutathione to the oocytes. Thus, they are required for cytoplasmic maturation and growth. Therefore, poor growth and development of cumulus cells has a negative effect on oocyte maturation and quality (26). In a study it was demonstrated that follicular selenium deficiency reduced glutathione peroxidase-1 expression. These findings suggest that selenium supplementation can affect the high oxidative stress levels experienced by women with PCOS and may also reduce the ovulation issues that cause infertility (27).

The results of a study in 2011 showed that there was a positive association between high selenium concentration and the number of follicles and oocyte function after ovarian stimulation, and a positive effect on ovarian response to gonadotropin therapy in IVF (28). Another study reported that selenium had a positive effect on increasing antioxidant capacity and women who received 200 mg/day of selenium had a higher fertility rate compared to the placebo group. A negative association was observed between selenium levels and androgenic hormones (12). Recently in China, a research team investigated the effects of selenium supplementation on meiosis, DNA integrity, oocyte growth, glutathione peroxidase activity in the oocyte, and expression of selenium-related genes in yak oocytes. They found that higher selenium concentrations were beneficial for yak oocyte maturation during in vitro maturation (29). These results are in line with our observations, and emphasize the need for appropriate selenium concentrations for oocyte growth and quality. In fact, in our study, by reducing the selenium concentration < 40 μg/ml, mature oocytes were affected and reduced. Therefore, while oogenesis is strongly related to intra-ovarian factors, especially FF factors, any imbalance or dysfunction between the internal and external ovarian factors may lead to abnormal folliculogenesis and impaired oogenesis (30).

Another study showed that increasing maternal age is associated with oocyte membrane abnormalities which occur due to decreases in fertility (31). At higher maternal ages, there is an increased frequency of meiotic errors, which significantly affects human oocytes. Because both meiotic and developmental competency are acquired in the late stages of oocyte growth, age-related defects in folliculogenesis will be effective in reducing oocyte quality (32). On the other hand, in a study conducted at Oxford University, it was hypothesized that differences in the oocyte-maturing gene expression occur in older women than in youngers ones; however,the results showed that the expression of MII and GV oocytes did not change with age (33). These results are in line with the results of our study which showed that the number of immature GV oocytes was higher in older women. Also, our results indicated that mature MII oocytes in women aged 35 yr and older was significantly lower than in younger women. Furthermore, in women aged ≥ 40 yr, the total number of oocytes and MI oocytes was lower than in women < 40 yr and the number of GV oocytes was significantly higher in 35-yr-old women than in younger ones. Our results are consistent with the aforementioned studies and so it can be concluded that the increasing age of women affects oocyte quantity in women with PCOS.

The results of another study showed that losing at least 5% of body weight improved metabolism and had a positive effect on ovulation and pregnancy in women with PCOS. Also, evidence shows that to improve menstruation and pregnancy rates, and lower testosterone levels, BMI should be < 27 (kg/m${}^{2}$) (34). In the present study, it was observed that the risk of anovulation increased with increasing BMI. In addition, another study conducted in 2013 showed that overweight and obese women with PCOS needed more gonadotropin injections and longer days of ovarian stimulation to reach follicular maturity than women with lower BMI but had fewer fertilized oocytes and good-quality embryos. This may be due to the fact that the quality of retrieved oocytes in overweight and obese women is worse than that in normal-weight women (35). On the other hand, another study reported that in women with PCOS undergoing IVF, despite the need for less gonadotropin stimulation, more oocytes were obtained, but these oocytes were less able to grow, were unable to complete meiosis, and were less capable of fertilization and normal embryo formation. This condition seemed to lead to more fat-burning than in normal individuals; women with PCOS produced more free radicals by burning further fats which can also affect the ovaries through oxidative stress and suppressed oocyte developmental competence (7). These results are consistent with the present study, which indicated that women with BMI ≥ 30 (kg/m${}^{2}$) had a significantly lower number of total oocytes, as well as in MI and MII oocytes. However, BMI did not show a significant association with difference in the number of GV oocytes.

In our study, having an infertility history of 5-10 yr was associated with a significantly lower total number of oocytes and an infertility duration of > 10 yr was associated with a remarkably lower number of MII mature oocytes. However, no considerable relationship was found between the number of immature MI oocytes and the duration of infertility.

One of the limitations of this study was the small sample size. A larger sample could help to provide more robust evidence of a correlation between selenium concentration in the FF and the quantity and maturity of oocytes. Also, this study failed to measure the concentration of selenium in the serum and the FF at the same time. This could have helped to determine the sufficient concentration of selenium in the FF.

## 5. Conclusion

According to the present study, low levels of follicular selenium concentration in infertile women with PCOS are associated with the number, maturity, and quality of oocytes taken from their ovaries. Therefore, it can be concluded that by reducing the FF selenium concentration, the oocyte quantity and maturation are likely to decrease.

##  Conflict of Interest

The authors declare they have no conflict of interest.
